# Validity of a practitioner-administered observational tool to measure physical activity, nutrition, and screen time in school-age programs

**DOI:** 10.1186/s12966-014-0145-5

**Published:** 2014-11-28

**Authors:** Rebekka M Lee, Karen M Emmons, Cassandra A Okechukwu, Jessica L Barrett, Erica L Kenney, Angie L Cradock, Catherine M Giles, Madeleine E deBlois, Steven L Gortmaker

**Affiliations:** Harvard School of Public Health, Social and Behavioral Sciences, 677 Huntington Avenue, Boston, MA 02115 USA; Kaiser Foundation Research Institute, Oakland, California

**Keywords:** Nutrition, Physical activity, Children, Screen time, Afterschool, Out-of-school time, Validation, Measurement

## Abstract

**Background:**

Nutrition and physical activity interventions have been effective in creating environmental changes in afterschool programs. However, accurate assessment can be time-consuming and expensive as initiatives are scaled up for optimal population impact. This study aims to determine the criterion validity of a simple, low-cost, practitioner-administered observational measure of afterschool physical activity, nutrition, and screen time practices and child behaviors.

**Methods:**

Directors from 35 programs in three cities completed the Out-of-School Nutrition and Physical Activity Observational Practice Assessment Tool (OSNAP-OPAT) on five days. Trained observers recorded snacks served and obtained accelerometer data each day during the same week. Observations of physical activity participation and snack consumption were conducted on two days. Correlations were calculated to validate weekly average estimates from OSNAP-OPAT compared to criterion measures. Weekly criterion averages are based on 175 meals served, snack consumption of 528 children, and physical activity levels of 356 children.

**Results:**

OSNAP-OPAT validly assessed serving water (r = 0.73), fruits and vegetables (r = 0.84), juice >4oz (r = 0.56), and grains (r = 0.60) at snack; sugary drinks (r = 0.70) and foods (r = 0.68) from outside the program; and children’s water consumption (r = 0.56) (all p <0.05). Reports of physical activity time offered were correlated with accelerometer estimates (minutes of moderate and vigorous physical activity r = 0.59, p = 0.02; vigorous physical activity r = 0.63, p = 0.01). The reported proportion of children participating in moderate and vigorous physical activity was correlated with observations (r = 0.48, p = 0.03), as were reports of computer (r = 0.85) and TV/movie (r = 0.68) time compared to direct observations (both p < 0.01).

**Conclusions:**

OSNAP-OPAT can assist researchers and practitioners in validly assessing nutrition and physical activity environments and behaviors in afterschool settings.

**Trial registration:**

Phase 1 of this measure validation was conducted during a study registered at clinicaltrials.gov NCT01396473.

**Electronic supplementary material:**

The online version of this article (doi:10.1186/s12966-014-0145-5) contains supplementary material, which is available to authorized users.

## Introduction

Public health researchers and practitioners have made the goals of increasing physical activity and improving healthy eating among youth a major national priority, recently through Michelle Obama’s Let’s Move Campaign [1]. These objectives are important given the rapid increase in childhood obesity in the United States, especially among Black and Latino children and those from economically disadvantaged backgrounds [2]. While current guidelines recommend children be physically active for at least 60 minutes each day, less than half of American children meet these standards [3-5]. Additionally, children seldom meet national dietary guidelines [6] and consumption of foods and drinks such as unhealthy snacks and sugary drinks has increased in recent years [7]. High obesity rates, low physical activity levels, and poor nutrition in childhood are all risk factors for chronic disease and higher obesity rates in adulthood [1].

Some of the most impactful strategies for improving youth activity and diet aim to create changes in school and afterschool environments. Interventions targeting these environments have been effective at increasing children’s physical activity and fitness [8-12], improving foods and beverages served and consumed by children [13-15], and decreasing obesity prevalence [13]. However, measuring physical activity and dietary intake with “gold standard” measures such as weighed plate waste and accelerometry requires training, time, and a substantial cost burden [16,17]. By contrast, lower cost surveillance measures for assessing trends in children’s physical activity and diet have typically relied on child and parent reports, which are subject to recall bias and have limited evidence for criterion validity [18-20]. While observational protocols have been developed for researchers to assess nutrition and physical activity among children in educational settings with less time and cost burden than “gold standard” measures [21,22], there has been limited focus on developing valid measures that practitioners can use. There is a need for tools teachers or afterschool staff can administer to assess program performance and evaluate progress as interventions are implemented. Measures that do not rely on trained research staff are also valuable for ongoing surveillance to simply and accurately track trends in program practices and child outcomes over time. In this paper, we aim to determine if a simple, low-cost, program-level, observational measure completed by afterschool program staff can validly assess afterschool physical activity and nutrition practices and child behaviors.

## Methods

### Design

We conducted this study in two phases. In phase 1, we tested the criterion validity of the observational practice assessment tool (OPAT) at follow-up data collection of the Out-of-School Nutrition and Physical Activity (OSNAP) randomized controlled trial in spring 2011 [15]. Data were collected in 20 Boston afterschool programs, 10 of which participated in the OSNAP nutrition and physical activity intervention. After the criterion validity of the original items was assessed, we tested nine revised items among 15 programs in two other Massachusetts cities in fall 2012 (Phase 2).

During both phases, afterschool staff completed OSNAP-OPAT during one week (up to five weekdays), and trained research staff collected concurrent criterion observational and physical activity monitor data. Program directors completed a written informed consent form and received $25 as compensation for completing the protocol. Parents (or guardians) completed written informed consent forms for collection of individual-level child data. The study was approved by the Harvard School of Public Health Office of Human Research Administration.

The OSNAP-OPAT items were focused on measuring the 10 OSNAP intervention goals: provide all children with at least 30 minutes of moderate to vigorous physical activity every day; offer 20 minutes of vigorous physical activity 3 times per week; do not serve sugary drinks; do not allow sugary drinks to be brought in during program time; offer water as a drink at snack every day; offer a fruit or vegetable option every day at snack; when serving grains (like bread, crackers, and cereals), serve whole grains; do not serve foods with trans fat; limit computer and digital device time to homework or instructional only; and eliminate use of commercial broadcast and cable TV and movies.

### Measures

#### Program characteristics

Parents reported child race/ethnicity, age, and gender on the consent form. Attendance was recorded during each day of data collection to determine program size. We obtained the proportion of students who were eligible for free or reduced price meals at the school hosting the program (or the school nearest the program) from administrative records. In phase 1, directors reported their years of experience, age, gender, education, hours of employment, race/ethnicity, and the number of staff at the program on a questionnaire.

#### Out-of-school nutrition and physical activity observational practice assessment tool (OSNAP-OPAT)

Study staff designed a paper and pencil daily observational practice assessment tool to align with the OSNAP intervention goals, which included items on physical activity, foods, beverages, and screen time (See Additional file 1 for complete tool). Two research assistants and two afterschool directors piloted the tool during afterschool program time, reviewing the usefulness of the instructions and glossary as well as the clarity of question wording and feasibility of completion. They reported that completing the OSNAP-OPAT for the program took 5–10 minutes to complete each day. Research assistants introduced OSNAP-OPAT to program directors before the program began on the Monday of each data collection week. Directors were instructed to answer the questions to the best of their ability based on what happened during the afterschool day. They were given a glossary with key physical activity and nutrition terms and a page for notes to gather input from other staff (Additional file 1).

In phase 1, OSNAP-OPAT was completed on five consecutive days by directors at 20 programs. Six items on program-provided foods and beverages served and three items on program screen time had dichotomous (yes/no) response categories. For each food or beverage offered, consumption was measured on a four-point scale. Two items on foods and beverages brought in from outside the snack program had four response categories corresponding to the number of children who consumed them. Five physical activity items measured the amount of time offered to children with dichotomous (yes/no) response categories for each question, while one item on the proportion of children participating in physical activity had five response categories. In phase 2, new items were fielded at 15 different programs. We tested six revised OSNAP-OPAT items on children’s physical activity with five response categories and three new dichotomous questions about serving sugar-sweetened beverages, 100% juice, and 100% juice >4oz.

#### Criterion measures

##### Physical activity

Child physical activity levels during the afterschool period were measured by Actigraph accelerometer (Models GT1M, GT3X, and GT3X+), considered a reliable and valid criterion measure for physical activity (see Table 1) [17,23,24]. Research assistants distributed accelerometers to participants on each day of data collection. During phase 1, accelerometer data were collected on five days (Monday-Friday); in phase 2, programs were typically offered on fewer days and, thus, accelerometer data were collected on three-five days. Accelerometers were fastened on the hip with an adjustable belt and children were instructed to wear them for the entire afterschool program period, except during swimming. We converted accelerometer intensity counts per one-minute epoch to total minutes of moderate and vigorous physical activity (MVPA) and vigorous physical activity (VPA) (i.e., including every minute above the corresponding threshold) during the afterschool period according to methods used in analysis of national surveillance data, using cut points and algorithms adapted from Troiano and colleagues [4,10].

**Table 1:** **Criterion measures used to validate the Out-of-School Nutrition and Physical Activity Observational Practice Assessment Tool (OSNAP-OPAT)**

**Measure**	**Weekly mean (SD)**	**Psychometric properties**	**Phase**	**Days**	**Corresponding OSNAP-OPAT items**
Minutes of moderate and vigorous physical activity per day via Actigraph accelerometer from all valid days^a^	15.30 (8.37)	High reliability (ICC = 0.80) over 4 days for children’s overall physical activity [23]	1 & 2	5	How many minutes do you think the typical child at your program was physically active today?
		Evidence for validity of children’s physical activity counts compared to doubly labeled water and VO_2_ max [17,24,25]			What is the least amount of physical activity time that was offered to any group of children today?
Minutes of vigorous physical activity per day via Actigraph accelerometer from all valid days^a^	1.97 (1.27)	High reliability (ICC = 0.80) over 4 days for children’s overall physical activity [23]	1 & 2	5	How many minutes do you think the typical child at your program was engaged in vigorous physical activity (i.e. activity more than a walk) today?
		Evidence for validity of children’s physical activity counts compared to doubly labeled water and VO_2_ max [17,24,25]			What is the least amount of vigorous physical activity time that was offered to any group of children today?
Percent of children engaged in moderate or vigorous physical activity during physical activity time via SOPLAY observations	68.60 (11.02)	High interrater reliability r = 0.76-0.98 [26]	1	2	How many children do you think were active when they attended physical activity time?
		Moderately valid compared to accelerometer estimates for boys and girls combined r = 0.40-0.58 [27]			
Direct observation of proportion of days items served at snack, including brand and size linked with nutrient database	FV: 0.56 (0.23)	Direct observation is a commonly used criterion measure for nutrition studies of school meals [28]	1	5	Was a fruit or vegetable offered at snack?
	Grain: 0.86 (0.24)				Were grains served at snack?
	Whole grain: 0.29 (0.24)				If grains were served at snack, were whole grains served?
	Water: 0.51 (0.43)				Was water served (with a pitcher or from a cooler) at snack?
	Juice: 0.36 (0.30)				Was 100% juice served at snack?
	Juice >4oz: 0.29 (0.33)				If 100% juice was served at snack, was it served in a container greater than 4oz?
Proportion of snack component(s) consumed according to direct observation and photography (Coded None 0, some 1, most 2, all 3)	Water: 0.14 (0.23)	High interrater reliability ICC = 0.78-0.92 [29]	1 & 2	2	For the children who were served water, how much do you think they drank?
		Valid in comparison to weighed plate waste protocol			
		Food items ICC = 0.86-0.94			
		Self-served water ICC = 0.47-0.52 [29]			
Direct observation of proportion of days screen time offered during the afterschool program	TV: 0.03 (0.08)	High interrater reliability of young children’s time watching television r = 0.96 [30]	1	5	Did your program offer any recreational (i.e. internet, entertainment) computer time today?
	Computer: 0.43 (0.40)				Did your program show any broadcast or cable TV or movies today?
Direct observation of number of children per day consuming foods and beverages brought in from outside the program snack	Sugary drinks: 2.11 (2.09)	High interrater agreement (87%) for item identification in elementary students’ home-packed lunches [31]	1	5	How many kids consumed sugary drinks from outside the snack program (e.g. vending, home, etc.) during the afterschool day?
	Food: 9.86 (12.20)				How many kids consumed food from outside the snack program during the afterschool day?

^a^Minutes of physical activity accumulated overall (i.e., counting every minute over the activity level threshold), computed according to methods used in analysis of national surveillance data, using Freedson cut points and algorithms adapted from Troiano and colleagues [4].

We assessed child participation in physical activity with structured observations according to a modified System for Observing Play and Leisure Activity in Youth (SOPLAY) protocol during all physical activity periods on two days at each program [26,27]. At two-minute intervals, research assistants rotated through physical activity spaces scanning from left to right and using a counter to tally the number of children engaged in sedentary, moderate, or vigorous physical activity.

##### Nutrition

The criterion measure for all nutrition outcomes was direct observation by trained observers (see Table 1) [28,29,31]. Research assistants recorded all food and beverage items served at snack, along with size, type, and brand, on 5 days at each program. Snack consumption of all consented children was recorded via direct observation and digital photography on two days at each program. Data collectors assessed whether children received each of the snack components and how much of each food or drink they consumed (e.g. none, some, most, or all). This observation measure was highly correlated with a weighted plate waste protocol [29]. The number of children who consumed foods and beverages from outside the program snack (e.g. from home, vending machines) were also recorded each day.

We obtained nutrition information for all foods and beverages observed from Boston Food and Nutrition Services, manufacturer’s websites, or from similar product listings in the US Department of Agriculture (USDA) Nutrient Database. Classification of foods and beverages was based the OSNAP intervention goals. Water served refers to water that was distributed as part of the program snack either via pitchers or coolers and cups in the snack area. It does not include water the children drank from water fountains or coolers outside the snack area or period. Sugary drinks were defined as beverages with added sugar and 100% juice greater than 4oz. Foods with trans fats were defined as items containing “partially hydrogenated” oil on ingredients list. Whole grains were defined as foods containing a whole grain as the first ingredient. Fruits and vegetables included any fresh, frozen, canned, or dried produce. Grain products included breads, cereals, crackers, etc.

##### Screen time

Research assistants completed a minute-by-minute log of each program day that included the number of minutes children were offered screen time (e.g. computers; television/movies).

### Statistical analysis

We summarized daily estimates of all OSNAP-OPAT items and criterion measures into weekly averages for each program. Data from 5 days of observations were used except for cases where fewer days were observed using the criterion measures (e.g. SOPLAY, snack intake) or programs ran on fewer than 5 days per week. To assess the criterion validity of OSNAP-OPAT, we calculated Pearson correlations comparing the weekly averages estimated by the OSNAP-OPAT to corresponding estimates from the criterion measures described in the methods and in Table 1. Criterion validity is the extent to which a measure performs like a “gold standard” measure, under the assumption that prediction, not explanation, indicates accuracy [20]. Correlations between 0.40 and 0.60 were considered moderate; those over 0.60 were considered strong.

All analyses were performed in 2013 using SAS 9.3 (SAS Institute, Cary, NC). For physical activity outcomes, we compared weekly average estimates from OSNAP-OPAT to weekly averages of SOPLAY observation data and accelerometer estimates. Midpoints of ranges in OSNAP-OPAT physical activity 5-point scales were used when computing minutes per day; for the 60+ minute category, 67.5 was used (see Additional file 1 for scale). Three days when swimming was offered and accelerometers were taken off were excluded from activity analyses. For nutrition outcomes, we compared weekly average estimates from OSNAP-OPAT to data collected from snack observations and the plate waste protocol. Weekly averages for screen time and outside food and beverage items from OSNAP-OPAT were compared to weekly averages from direct observations. We conducted linear regressions to determine if intervention status in phase 1 was associated with the accuracy of responses.

For those OSNAP-OPAT items with at least a moderate correlation among weekly estimates (r>/=0.40), we calculated Pearson correlations using only one day of OSNAP-OPAT data to predict average weekly criterion outcomes. For each day of the week (Monday-Friday), daily OSNAP-OPAT estimates were compared to the average weekly criterion measure, and the average Pearson correlations across the days were calculated.

## Results

During phase 1, directors from 18 programs completed the practice assessment on five days each. One program completed OSNAP-OPAT on four days and another on only three days. In phase 2, most programs ran Monday-Thursday; thus, practice assessment and criterion data were usually collected on four days. Table 2 shows characteristics of the afterschool programs and the children who attended the programs. The typical program served about 50 children each day. Programs in this sample served more children of color (78%) than white children (22%) and 80% students at the school where the programs were based qualified for free/reduced price lunch.

**Table 2:** **Characteristics of afterschool programs and children where the Out-of-School Nutrition and Physical Activity Observational Practice Assessment Tool (OSNAP-OPAT) was completed**

**Program characteristics**_**a**_	**Phase 1 (N = 20)**	**Phase 2 (N = 15)**
Average daily attendance per site, mean (SD)	55.0 (49.9)	44.6 (17.9)
*Sponsoring agency, n (%)*		
YMCA	8 (40.0%)	2 (13.3%)
Boys & Girls Club	4 (20.0%)	0 (0.0%)
Community Centers	5 (25.0%)	3 (20.0%)
School	3 (15.0%)	10 (66.7%)
Proportion of students at the school where the program is based who qualify for free or reduced price meals	81.6%	79.2%
**Child characteristics**_**b**_	**Phase 1 (N = 596)**	**Phase 2 (N = 389)**
Age (yrs.), mean (SD)	7.8 (1.8)	8.6 (1.8)
Male, (%)	50.8%	50.7%
*Race/ethnicity, (%)*		
White	9.2%	34.2%
Black/African American	29.5%	5.4%
Hispanic/Latino	29.7%	31.1%
Asian	4.2%	1.3%
Cape Verdean	2.7%	1.6%
Black Hispanic	3.2%	0.0%
Multiracial	4.9%	14.0%
Missing	16.6%	12.4%

a) The average daily attendance was recorded at each site by research assistants during the week of data collection. The proportion of children who qualify for free or reduced price meals was collected via administrative records. Programs that were non-school based used data from the closest school as a proxy.

b) Child characteristics were reported by parents on consent forms.

Directors who completed OSNAP-OPAT in phase 1 had an average of 3.5 (SD = 4.3) years in their role and an average age of 36 (SD = 10.6). Thirteen of 20 directors were women and 16 had a college degree. On average, the directors worked at the program 33 hours per week (SD = 9.4); some worked as full-time benefited employees, while other worked part-time.

### Phase 1

Pearson correlations summarizing the criterion validity of each OSNAP-OPAT item are reported in Table 3. Reported physical activity participation levels were correlated with estimates of the proportion of children participating in MVPA via SOPLAY (r = 0.48, p = 0.03). Directors’ reports of serving water, fruits and vegetables, and grains at snack time were significantly correlated with observations by trained data collectors (r = 0.73, r = 0.84, r = 0.60 respectively; all p < 0.01). There was a moderate correlation between reports and observations of whole grains being served (r = 0.43, p = 0.06). Additionally, directors validly reported how much children drank when they were served water compared to direct observations (r = 0.56, p = 0.01). The reported numbers of children who consumed sugary drinks and snacks brought in from outside the snack program were correlated with the number recorded in daily observations (r = 0.70, r = 0.68 respectively; both p < 0.01). Directors’ reports of offering recreational computer time and showing TV or movies were significantly correlated with observations by trained data collectors (r = 0.85, r = 0.68 respectively; both p < 0.01). Participating in a nutrition and physical activity intervention did not impact accuracy of any OSNAP-OPAT responses. When one day of OSNAP-OPAT data was correlated with weekly average outcomes, correlations were attenuated as expected to varying degrees (Table 3) [20].

**Table 3:** **Criterion validity of the Out-of-School Nutrition and Physical Activity Observational Practice Assessment Tool (OSNAP-OPAT)**

**Item**	**N** ^**a**^	**Weekly mean (SD)** ^**b**^	**Validation criterion measure**	**Pearson correlation (week)** ^**b**^	**P value**	**Pearson correlation (1 day)** ^**c**^	**Range**
**Physical Activity**							
How many children do you think were active when they attended physical activity time? (0–4 scale: 0 Not offered, 1 Few, 2 Some, 3 Most, 4 All)	20	2.54 (0.39)	SOPLAY	0.48	0.03	0.28	0.09-0.50
How many minutes do you think the typical child at your program was physically active today? (Mins)	15	34.40 (12.31)	Accelerometer	0.43	0.11	0.23	−0.17-0.44
What is the least amount of physical activity time that was offered to any group of children today? (Mins)	15	21.13 (14.88)	Accelerometer	0.59	0.02	0.33	−0.19-0.62
How many minutes do you think the typical child at your program was engaged in vigorous physical activity (i.e. activity more than a walk) today? (Mins)	15	26.59 (13.46)	Accelerometer	0.43	0.11	0.21	0.09-0.36
What is the least amount of vigorous physical activity time that was offered to any group of children today? (Mins)	15	18.29 (14.41)	Accelerometer	0.63	0.01	0.42	0.13-0.61
**Nutrition**							
Was a fruit or vegetable offered at snack? (Proportion)	20	0.59 (0.29)	Observation	0.84	0.0001	0.52	0.38-0.66
Were grains served at snack? (Proportion)	20	0.68 (0.34)	Observation	0.60	0.005	0.47	0.25-0.56
If grains were served at snack, were whole grains served? (Proportion)	20	0.47 (0.32)	Observation	0.43	0.06	0.32	0.17-0.50
Was water served (with a pitcher or from a cooler) at snack? (Proportion)	20	0.70 (0.41)	Observation	0.73	0.0003	0.66	0.59-0.72
How many kids consumed sugary drinks from outside the snack program (e.g. vending, home, etc.) during the afterschool day? (0–3 scale: 0 None, 1 Few, 2 Some, 3 Many)	20	0.74 (0.78)	Observation	0.70	0.0005	0.62	0.54-0.70
How many kids consumed food from outside the snack program during the afterschool day? (0–3 scale: 0 None, 1 Few, 2 Some, 3 Many)	20	1.06 (0.65)	Observation	0.68	0.001	0.65	0.48-0.82
Was 100% juice served at snack? (Proportion)	15	0.29 (0.32)	Observation	0.50	0.06	0.42	0.17-0.82
If 100% juice was served at snack, was it served in a container greater than 4oz? (Proportion)	15	0.25 (0.31)	Observation	0.56	0.03	0.59	0.33-0.86
For the children who were served water, how much do you think they drank? (0–3 scale: 0 None, 1 Some, 2 Most, 3 All)	20	0.64 (0.36)	Plate waste	0.56	0.01	0.49	0.39-0.60
**Screen time**							
Did your program offer any recreational (i.e. internet, entertainment) computer time today? (Proportion)	20	0.39 (0.35)	Observation	0.85	0.0001	0.60	0.46-0.72
Did your program show any broadcast or cable TV or movies today? (Proportion)	20	0.03 (0.10)	Observation	0.68	0.0007	0.50	0.49-0.50

a. Criterion validity assessed in 20 programs during phase 1 and 15 programs during phase 2.

b. Pearson correlations for weekly OSNAP-OPAT data were based on 5 days of data in phase 1. In phase 2, data was collected on 4 days in 9 sites, 5 days in 3 sites, and 3 days in 3 sites.

c. Pearson correlations comparing one day of OSNAP-OPAT data to average weekly offerings via criterion measures were calculated for each day of the week and then averaged to get a validity estimate for one day of OSNAP-OPAT data.

In contrast, dichotomous OSNAP-OPAT items for offering 30 minutes or more of MVPA and 20 minutes or more of VPA were not correlated with accelerometer estimates. Serving foods with trans fats, serving sugary drinks, and limiting computer time to less than one hour per child were not correlated with observational criterion measures. No other OSNAP-OPAT reports of child consumption were significantly correlated with plate waste estimates (see Additional file 2 for items dropped from the original measure).

### Phase 2

When we revised OSNAP-OPAT items on children’s physical activity time from a dichotomous response to a five-point scale, correlations with accelerometer estimates improved. Minutes of MVPA were moderately correlated with director reported activity time for a typical child (r = 0.43, p = 0.11) and more strongly correlated with the minimum number of minutes directors reported offering any group during the program day (r = 0.59, p = 0.02). Similarly, minutes of VPA were moderately correlated with reported vigorous activity time for a typical child (r = 0.43, p = 0.11) and more strongly correlated with the minimum number of vigorous minutes directors reported offering any group during the program day (r = 0.63, p = 0.01). When we separated the OSNAP-OPAT sugary beverage assessment into three distinct questions, juice (r = 0.50, p = 0.06) and juice >4oz (r = 0.56, p = 0.03) were correlated with snacks observed. No beverages with added sugar were served.

The final OSNAP-OPAT tool is available in Additional file 1 and online at http://osnap.org/tools/practice-assessment/introduction/. We estimated that collecting data on Monday-Friday at a typical program costs about $13, including cost of paper and staff time to complete the assessment.

## Discussion

This study establishes the criterion validity of a brief, low-cost, program-level observational measure of nutrition, physical activity, and screen time in afterschool programs that can be implemented by practitioners. When completed by afterschool directors, OSNAP-OPAT validly assessed serving water, fruits and vegetables, grains, and juice at snack as well as the amount of sugary drinks and snacks brought in from outside the program. Directors also validly assessed children’s water consumption. Reported physical activity participation was correlated with the proportion of children observed participating in MVPA and the amount of physical activity time reported was correlated with MVPA and VPA accelerometer estimates. The instrument validly assessed whether computer and television or movie time was offered during the program. Using OSNAP-OPAT, program directors were able to accurately assess program and participant practices related to obesity risk factors—healthy beverages, screen time, physical activity, and fruits and beverages—which have become the core components of recommended evidenced-based community interventions and programs [1,3].

Findings from this multi-phase study highlight important measurement considerations. In phase 1, dichotomous questions on the amount of time physical activity offered were not correlated with accelerometer data since most programs offered the targeted 30 minutes of daily physical activity and little variation existed in responses. When we changed items to a scale with five time intervals, physical activity items were significantly correlated with corresponding accelerometer estimates. Afterschool directors’ reports of the least amount of physical activity offered to any group of children were more highly correlated with children’s objectively measured physical activity levels than their reports of the physical activity of a typical child at the program. In phase 2, splitting a single sugary beverage question into three distinct items on juice, large juice (>4oz), and beverages with added sugar yielded valid assessments.

To date, limited research has sought to develop valid nutrition and physical activity measures that are easy for practitioners to use in free-living settings like afterschool programs with limited training and at low cost [21]. However, researchers have begun creating measures designed for early childcare staff [32,33]. The current study builds on this evidence base by using strong criterion measures over the course of one week to obtain a more precise estimate of nutrition and physical activity practices and behaviors. It also uses repeated assessment data to determine how one day of data performs in comparison to weekly criterion measures. While some items were significantly correlated with the criterion with one day of data, as expected the ranges of these estimates were wide. Whenever possible, researchers and program staff should use weekly averages, rather than one-day estimates. OSNAP-OPAT is low cost and requires minimal staff time to complete. Plus, the instructions for utilizing the measure are built into the tool, rather than requiring the extra expense and time of training. Its user-friendly format can help afterschool staff take action to improve their current practices. The measure and companion tools for identifying areas for improvement and developing action plans are available for free at www.osnap.org.

This study has several limitations. OSNAP-OPAT items completed by directors could not accurately assess the serving of snacks with trans fats nor children’s consumption of foods and beverages other than water. These items have been removed from the final measure. We encourage future research on the best way to capture accurate data on these items from practitioners in the field. The generalizability of our findings is limited due to the small sample of programs all located in Massachusetts who agreed to participate in a nutrition and physical activity intervention. However, the instrument was tested in programs sponsored by a range of organizations and in low income, racially diverse settings. While we were able to establish criterion validity—the extent to which OSNAP-OPAT performs similarly to “gold standard” measures of trained direct observation and accelerometry, assessing the measure’s content and construct validity were outside the scope of this study. Finally, because we initialized accelerometers using one-minute epochs according to the guidelines from the National Cancer Institute to be consistent with methods used in national surveillance, we were unable to analyze shorter physical activity intervals that may have captured intermittent physical activity. Recent research indicates that the Actigraph accelerometers and one-minute intervals capture children’s energy expenditure well compared to room calorimeters and doubly labeled water methods [25].

## Conclusion

OSNAP-OPAT is a measure that can be used to validly assess program performance as interventions are implemented and evaluated in real world afterschool settings. This brief assessment tool will help researchers and practitioners gain an accurate assessment of afterschool program practices and child behaviors and target specific areas for improvement as they initiate and evaluate obesity prevention initiatives.

## Additional files

Additional file 1:
**Out-of-school Nutrition and Physical Activity (OSNAP) Observational Practice Assessment Tool (OPAT).**
Additional file 2:
**Items not retained in final tool.**

## Abbreviations

OSNAP-OPAT: Out-of-school nutrition and physical activity observational practice assessment tool
MVPA: Moderate and vigorous physical activity [the measure]
VPA: Vigorous physical activity
OSNAP: Out-of-School Nutrition and Physical Activity Initiative [the intervention]
USDA: United States Department of Agriculture
SOPLAY: System for Observing Play and Leisure Activity in Youth
FV: Fruit and vegetable
SD: Standard deviation
N: Number
Mins: Minutes
ICC: Intraclass correlation
R: Correlation coefficient
Yrs: Years
