# Supplementary material for: Validity of a practitioner-administered observational tool to measure physical activity, nutrition, and screen time in school-age programs
Source: Int J Behav Nutr Phys Act. 2014 Nov 28;11:145. doi: 10.1186/s12966-014-0145-5 (PMC4264534; doi:10.1186/s12966-014-0145-5)
Supplement: Additional file 1: — Out-of-school Nutrition and Physical Activity (OSNAP) Observational Practice Assessment Tool (OPAT). [file 12966_2014_145_MOESM1_ESM.doc]

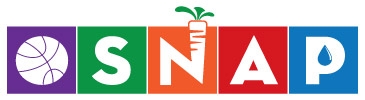
**Out-of-school Nutrition and Physical Activity (OSNAP)**

**Observational Practice Assessment Tool (OPAT)**

This observation tool can be a valuable resource for assessing afterschool environments. When completing the tool, it is important to honestly assess what you see during the afterschool day so that the results can be used to help you set and meet goals for improving nutrition and physical activity practices. You should make sure to observe children at snack time and when they are physically active. It should take 5-10 minutes to fill out the form at the end of the day.

This tool is being used as part of a research study to determine children’s physical activity and nutrition in out-of-school settings. Completing the tool is voluntary. If you have any questions as you are completing it, please contact Rebekka Lee at 617-384-5457.

**Getting started:** Before you begin, take some time to plan how you can best observe the physical activity and eating practices and behaviors of the children in your program. You may not be able to see *everything* that’s going on in the program, but this planning will ensure you prioritize when to be where. It is important that the person who completes this assessment can observe all or most of the snack and physical activity time during your program. Review the questions and the glossary to make sure you know what information you’ll need to report on. Also, make sure to consider the following questions as you plan your self-assessment:

- *When is physical activity offered?*  *Is it offered at different times or all at once?*
- *Do kids break out into different groups for physical activity? If so, where in the building do these groups meet?*
- *When is snack served? Do all the kids eat together or at different times?*

If you have a schedule of the program day you may want to use it to plan out your observations. But, do not use your schedule to fill in this tool. What you record should be a measure of what actually happened during the afterschool day.

**Instructions:** Complete this self-assessment tool each day, Monday through Friday, to get the best assessment of our program offerings. Make sure to answer all questions on the form by the end of each day. It is important that you answer the questions to the best of your ability. If the children in your program split into groups, work with group leaders to make sure to accurately report the activities and participation of all children at your site. Feel free to use the notes section on page 5 to keep track of what you see during the day.

**OSNAP Nutrition and Physical Activity Glossary of Terms**

**Physical activity** is any activity more than sitting or standing (includes activities such as walking, stretching, running, throwing, etc.).

**Vigorous physical activity** is any activity more than a walk (includes activities such as playground free play, jogging, swimming, etc.).

**Groups of children** are those that are formally designated by the program, such sites that break children into groups by age, grade, gender etc. for activities. Questions that refer to groups of children do NOT mean smaller, more informal friend groups etc.

**Screen Time** includes time watching television and DVDs, using computers, and playing video games.

**Commercial Broadcast TV/Movies** is any screen time shown primarily for entertainment purposes; this includes shows or movies without commercials but shown for entertainment purposes.

**Recreational Computer Use** is time on the computer primarily for entertainment purposes. Any time on the internet is considered recreational computer use. In contrast, educational computer use is use of computers that emphasizes academics & formal instruction, such as games that promote acquisition of math skills or use of word processing application to write a paper or story.

**Sugary drinks** include soda, sweetened ice teas, fruit punches, fruit drinks, sports drinks, sweetened water (e.g. Vitamin water), and any juice greater than 4 ounces (a half a cup) in size.

**Whole grains** are foods that contain a whole grain as the first ingredient on the label. Examples of whole grains are whole wheat, whole corn, barley, oats, and rye.

**Water served** refers to water that is distributed as part of the program snack, either via pitchers, a cooler/Cambro in the snack area, or in bottles. This does NOT include water children drink from a water fountains or from coolers outside of the snack room/period.

**Outside drinks and food** are those items that are brought in from home, outside restaurants or convenience stores, purchased from vending machines on site, or distributed by program partners during activity outside of the snack period. This would include any food or drink that is not part of the afterschool snack program.

**Physical Activity & Screen time**

1. How many minutes do you think the typical child at your program was physically active today?

🞏 0 minutes 🞏 1-14 minutes 🞏 15-29 minutes 🞏 30-44 minutes 🞏 45-59 minutes 🞏 60 minutes or more

2. Did your program offer any physical activity time today (for example, free play outside or sports/dance programming)? 🞏 Yes 🞏 No

3. What is the most amount of physical activity time that was offered to any group of children today?

🞏 No PA offered 🞏 1-14 minutes 🞏 15-29 minutes 🞏 30-44 minutes 🞏 45-59 minutes 🞏 60 minutes or more

4. What is the least amount of physical activity time that was offered to any group of children today?

🞏 No PA offered 🞏 1-14 minutes 🞏 15-29 minutes 🞏 30-44 minutes 🞏 45-59 minutes 🞏 60 minutes or more

5. How many children do you think were active when they attended physical activity time?

🞏 No PA offered 🞏 None to 1/4 of kids 🞏 More than 1/4 to half of kids 🞏 More than half to 3/4 of kids 🞏 More than 3/4 to all kids

6. How many minutes do you think the typical child at your program was engaged in vigorous physical activity (i.e. activity more than a walk) today?

🞏 0 minutes 🞏 1-9 minutes 🞏 10-19 minutes 🞏 20-29 minutes 🞏 30-59 minutes 🞏 60 minutes or more

7. Did your program offer any vigorous physical activity time today? 🞏 Yes 🞏 No

8. What is the most amount of vigorous physical activity time that was offered to any group of children today?

🞏 No vigorous PA offered 🞏 1-9 minutes 🞏 10-19 minutes 🞏 20-29 minutes 🞏 30-59 minutes 🞏 60 minutes or more

9. What is the least amount of vigorous physical activity time that was offered to any group of children today?

🞏 No vigorous PA offered 🞏 1-9 minutes 🞏 10-19 minutes 🞏 20-29 minutes 🞏 30-59 minutes 🞏 60 minutes or more

10. Did your program show any broadcast or cable TV or movies today? 🞏 Yes 🞏 No

11. Did you see any children using electronic hand-held devices (for uses other than homework/instruction)? 🞏 Yes 🞏 No

12. Did your program offer any recreational (i.e. on the internet, entertainment) computer time today? 🞏 Yes 🞏 No

13. How much recreational computer time was allowed for each child?

🞏 No computer time 🞏 1-14 minutes 🞏 15-29 minutes 🞏 30-44 minutes 🞏 45-59 minutes 🞏 60 minutes or more

**Nutrition**

14. Was a fruit or vegetable served at snack? 🞏 Yes 🞏 No

15. Were any grains served at snack? 🞏 Yes 🞏 No

16. If grains were served at snack, were they snacks that contain a whole grain as the first ingredient?

🞏 NA (no grains served) 🞏 Yes (whole grains served) 🞏 No (grain served but not a whole grain)

17. Were drinks with sugar added like soda, sweetened teas, fruit punches, or sports drinks served at snack? 🞏 Yes 🞏 No

18. Was 100% juice served at snack? 🞏 Yes 🞏 No

19. If 100% juice was served at snack, was it served in a container greater than 4oz?

🞏 NA (no juice served) 🞏 Yes (>4oz of juice was served) 🞏 No (juice was served in a small container)

20. Was water served (with a pitcher or from a cooler) at snack? *If no, skip to question 22.* 🞏 Yes 🞏 No

21. For ***the children who were served water***, how much do you think they drank?

🞏 None 🞏 Some 🞏 Most 🞏 All

22. How many kids consumed sugary drinks from outside the snack program (e.g. vending, home, etc.) during the afterschool day?

🞏 None 🞏 Few (1-5 kids) 🞏 Some (6-10 kids) 🞏 Many (>10 kids)

23. How many kids consumed food from outside the snack program during the afterschool day?

🞏 None 🞏 Few (1-5 kids) 🞏 Some (6-10 kids) 🞏 Many (>10 kids)

**Notes Page**

This notes page is a place for you to keep track of any important nutrition or physical activity related observations you make during the afterschool day. You may find it useful to jot down things like the start and end times of physical activity, tallies of kids you see eating or drinking outside food etc. This can be a good reference for accurately filling out the self-assessment at the end of the day.

Things to remember about physical activity…

Things to remember about computer, TV or movies…

Things to remember about outside foods and drinks...

Things to remember about food and drinks at snack…
