# Supplementary material for: Validity of a practitioner-administered observational tool to measure physical activity, nutrition, and screen time in school-age programs
Source: Int J Behav Nutr Phys Act. 2014 Nov 28;11:145. doi: 10.1186/s12966-014-0145-5 (PMC4264534; doi:10.1186/s12966-014-0145-5)
Supplement: Additional file 2: — Items not retained in final tool. [file 12966_2014_145_MOESM2_ESM.docx]

**Additional File 2 – Items Not Retained in Final Tool**

| **Items** | **Scoring** | **Decision Point** |
| --- | --- | --- |
| Did your program offer 30 or more minutes of physical activity to any group of children? | 0 = No  1 = Yes | Revised |
| Did all groups of children attend 30 or more minutes of physical activity? | 0 = No  1 = Yes | Revised |
| Were 20 minutes or more of vigorous physical activity offered? | 0 = No  1 = Yes | Revised |
| Was recreational computer time limited to under 1 hour per child? | 0 = No  1 = Yes | Dropped |
| For the children who were served a fruit or vegetable, how much do you think they ate? | 0 = None  1 = Some  2 = Most  3 = All | Dropped |
| Were foods with trans fats (i.e. items with “partially hydrogenated oil” served at snack? | 0 = No  1 = Yes | Dropped |
| For the children who were served this grain item, how much do you think they ate? | 0 = None  1 = Some  2 = Most  3 = All | Dropped |
| Were sugary drinks (with sugar added or juice >4oz) served at snack? | 0 = No  1 = Yes | Revised |
| For the children who were served sugary drinks, how much do you think they drank? | 0 = None  1 = Some  2 = Most  3 = All | Dropped |
